# A Frameshift Mutation in the Cubilin Gene (*CUBN*) in Border Collies with Imerslund-Gräsbeck Syndrome (Selective Cobalamin Malabsorption)

**DOI:** 10.1371/journal.pone.0061144

**Published:** 2013-04-16

**Authors:** Marta Owczarek-Lipska, Vidhya Jagannathan, Cord Drögemüller, Sabina Lutz, Barbara Glanemann, Tosso Leeb, Peter H. Kook

**Affiliations:** 1 Institute of Genetics, Vetsuisse Faculty, University of Bern, Bern, Switzerland; 2 Clinic for Small Animal Internal Medicine, Vetsuisse Faculty, University of Zurich, Zurich, Switzerland; 3 Department of Veterinary Clinical Science, The Royal Veterinary College, University of London, North Mymms, Hertfordshire, United Kingdom; University of Sydney, United States of America

## Abstract

Imerslund-Gräsbeck syndrome (IGS) or selective cobalamin malabsorption has been described in humans and dogs. IGS occurs in Border Collies and is inherited as a monogenic autosomal recessive trait in this breed. Using 7 IGS cases and 7 non-affected controls we mapped the causative mutation by genome-wide association and homozygosity mapping to a 3.53 Mb interval on chromosome 2. We re-sequenced the genome of one affected dog at ∼10× coverage and detected 17 non-synonymous variants in the critical interval. Two of these non-synonymous variants were in the cubilin gene (*CUBN*), which is known to play an essential role in cobalamin uptake from the ileum. We tested these two *CUBN* variants for association with IGS in larger cohorts of dogs and found that only one of them was perfectly associated with the phenotype. This variant, a single base pair deletion (c.8392delC), is predicted to cause a frameshift and premature stop codon in the *CUBN* gene. The resulting mutant open reading frame is 821 codons shorter than the wildtype open reading frame (p.Q2798Rfs*3). Interestingly, we observed an additional nonsense mutation in the *MRC1* gene encoding the mannose receptor, C type 1, which was in perfect linkage disequilibrium with the *CUBN* frameshift mutation. Based on our genetic data and the known role of CUBN for cobalamin uptake we conclude that the identified *CUBN* frameshift mutation is most likely causative for IGS in Border Collies.

## Introduction

Cobalamin is a member of the B-group, water soluble vitamins. Cobalamin is also known as vitamin B_12_. The abbreviation B_12_ covers all forms of cobalamins (i.e. all compounds with a corrin ring structure) and not only cyanocobalamin, which is the vitamin B_12_ [1]. Higher organisms such as plants or animals are unable to synthesize cobalamin. Mammals rely on either dietary cobalamin or symbiontic microorganisms to obtain this essential compound. Vitamin B_12_ serves as coenzyme for 5-methyltetrahydrofolate-homocysteine methyltransferase (MTR) and methylmalonyl coenzyme A mutase (MUT). Deficiency of cobalamin leads to reduced activity of both of these enzymes resulting in an increase of methylmalonic acid (MMA) and total homocysteine (tHcy). Therefore, measurements of these metabolites allow the assessment of cellular cobalamin availability and are the tests of choice to detect early or mild cobalamin deficiency in humans [1].

MTR is involved in the synthesis of DNA. Therefore, a lack of cobalamin affects rapidly dividing cells and leads to changes in the hematopoietic system such as megaloblastic anemia. Chronic deficiency of cobalamin also leads to neurological symptoms and irreversible damage of the brain and nervous system [1].

The uptake of dietary cobalamin in mammals is a complex process requiring several endogenous proteins. In the small intestine the dietary cobalamin (“extrinsic factor”) is bound to a secreted protein, called gastric intrinsic factor (GIF). The uptake of the cobalamin-GIF complex from the intestinal lumen into the body is mediated by a specific membrane protein complex termed cubam receptor [2], [3]. Cubam consists of two separate protein subunits, amnionless (AMN) and cubilin (CUBN).

Mutations in either the *AMN* or *CUBN* genes lead to Imerslund-Gräsbeck syndrome (IGS) or selective cobalamin malabsorption [4], [5]. IGS in humans is a rare autosomal recessive disorder, which results in megaloblastic anemia, mild proteinuria, failure to thrive, and neurological damage when untreated. IGS can be successfully managed by supplementation with regular doses of cobalamin [6].

Primary cobalamin absorption disorders have also been reported in several dog breeds including Australian Shepherd Dogs [7], Beagles [8], Border Collies [9]–[12], Giant Schnauzers [13], and Shar-Peis [14]. Two independent mutations in the *AMN* gene cause IGS in Australian Shepherd Dogs and Giant Schnauzers, respectively [7]. The molecular defect in the other dog breeds has not yet been reported.

We previously described clinical and laboratory findings in young Border Collies with IGS and established breed-specific reference parameters for serum cobalamin, urine methymalonic acid, and plasma homocysteine concentrations in Border Collies [12], [15]. The goal of the present study was the identification of the causative mutation for IGS in the Border Collie breed.

## Results

### Phenotypic Description

We previously described the clinical phenotype of IGS in Border Collies in detail [12]. Briefly, affected dogs were young (median 11.5 months, range 8–42 months) when finally diagnosed. Historical complaints included intermittent diarrhea, inappetence (picky appetite to anorexia) with poor body condition (BCS 2–3/9), and failure to grow. Clinically the marked weakness and small growth were the predominant findings. Additional clinical signs included odynophagia, glossitis, and bradyarrhythmia. Pertinent laboratory abnormalities consisted of mild to moderate normocytic, non-regenerative anemia with evidence of dyserythropoesis (increased numbers of nucleated red blood cells), increased aspartate-aminotransferase activity, and mild proteinuria. All dogs had serum cobalamin levels below the detection limit of the assay, as well as marked methylmalonic aciduria and hyperhomocysteinemia. We achieved full clinical recovery in all dogs with regular parenteral cobalamin supplementation [12].

### Mapping of the Causative Mutation

**Figure 1:**
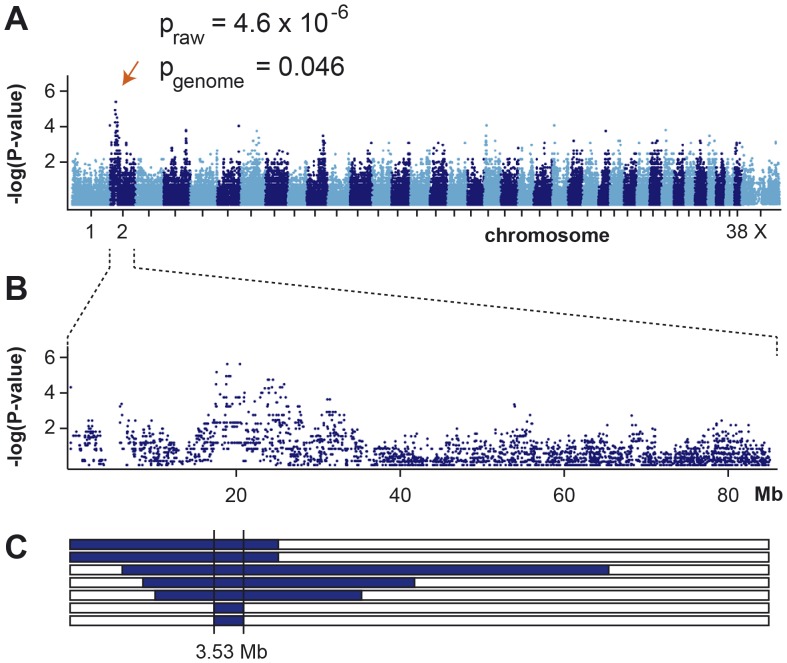
Mapping of IGS in Border Collies. (A) A genome-wide association study using 7 cases and 7 controls indicates a signal on CFA 2. (B) The detailed view of CFA 2 delineates an associated interval of ∼5 Mb. (C) Homozygosity mapping. Each horizontal bar corresponds to the CFA 2 genotypes of the 7 analyzed cases. Homozygous regions with shared alleles are shown in blue. A shared homozygous interval delineates the exact boundaries of the critical interval from 17,283,880 to 20,818,258 bp (CanFam 3 assembly).

We genotyped 173,662 evenly spaced SNPs on DNA samples from 7 affected Border Collies and 7 controls. After removing 61,339 markers, which had bad call rates (<90%), were non-informative (MAF <0.05), or showed a strong deviation from Hardy-Weinberg equilibrium in the controls (p<10^−5^), we retained 112,323 markers for the final genome-wide allelic association study. Three best-associated SNPs in the GWAS had identical raw p-values of 4.6×10^−6^ (Figure 1A). The corrected p-value after 100,000 permutations was 0.046. The 19 best-associated SNPs with raw p-values of less than 8×10^−5^ were all located on CFA 2 (Figure 1B). It has to be cautioned that the genomic inflation factor in this analysis was 1.36. This high value indicates sample stratification, which was caused by the use of closely related dogs including one full-sib pair among the cases. As the GWAS results showed an unequivocal signal we did not perform sophisticated corrections for the stratification in our samples.

Subsequently, we applied a homozygosity mapping approach to fine-map the region containing the disproportionate dwarfism mutation. We hypothesized that the affected dogs most likely were inbred to one single founder animal. Under this scenario the affected individuals were expected to be identical by descent (IBD) for the causative mutation and flanking chromosomal segments. We analyzed the cases for extended regions of homozygosity with simultaneous allele sharing. Only one genome region, which coincided with the associated interval on CFA 2, fulfilled our search criteria. Here, all 7 affected dogs were homozygous and shared identical alleles over 229 SNP markers corresponding to a ∼3.5 Mb interval. We concluded that the causative mutation should be located in the 3.53 Mb critical interval between the closest heterozygous markers on either side of the homozygous segment (CFA2∶17,283,880–20,818,258, CanFam 3 assembly; Figure 1C).

### Mutation Identification

A total of 33 genes and loci are annotated in the critical interval on CFA 2 (CanFam 3.1, Table S1). In order to obtain a comprehensive overview of all variants in the critical interval we sequenced the whole genome of one affected Border Collie. We collected 127 million 2×100 bp paired-end reads from a shotgun fragment library corresponding to roughly 10× coverage of the genome. We called SNPs and indel variants with respect to the reference genome of a presumably non-affected Boxer. Across the entire genome, we detected 2.5 million homozygous variants (Table 1). Within the critical interval there were 3,173 variants, of which 17 were predicted to be non-synonymous (Table S2). We further compared the genotypes of the affected Border Collie with 12 dog genomes of various breeds that had been sequenced in our laboratory in the course of other ongoing studies. We hypothesized that the mutant allele at the causative variant should be completely absent from all other dog breeds outside Border Collies as the large size of the associated haplotype clearly indicated a relatively young origin of the mutation. Therefore, we considered it unlikely that the mutant allele would have been introgressed into any other breeds outside Border Collies. Among the 17 non-synonymous variants, there were only 3 variants where the affected Border Collie carried the homozygous variant genotype and all other 12 sequenced dogs carried the homozygous wildtype genotype (Table 2).

**Table 1:** Variants detected by whole genome re-sequencing of an affected Border Collie.

Filtering step	Number of variants
Variants in the whole genome^a^	2,519,661
Variants in the critical 3.53 Mb interval on CFA 2	3,173
Variants in the critical interval that were absent from 12 other dog genomes	243
Non-synonymous variants in the whole genome^a^	68,303
Non-synonymous variants in the critical 3.53 Mb interval on CFA 2	17
Non-synonymous variants in the critical interval that were absent from 12 other dog genomes	3

a The sequences were compared to the reference genome (CanFam 3) from a Boxer. Only variants that were homozygous in the affected Border Collie are reported.

**Table 2:** Three non-synonymous variants in the critical interval of an affected Border Collie that were absent from 12 other dog genomes.

Position on CFA 2(CanFam 3 assembly)	Reference allele	Variant allele	Gene	Variant (cDNA)	Variant (protein)
19,125,603	G	A	*MRC1*	c.2143C>T	p.R715*
19,974,334	C	–	*CUBN*	c.8392delC	p.Q2798Rfs*3
19,999,374	G	C	*CUBN*	c.9215G>C	p.S3072T

**Figure 2:**
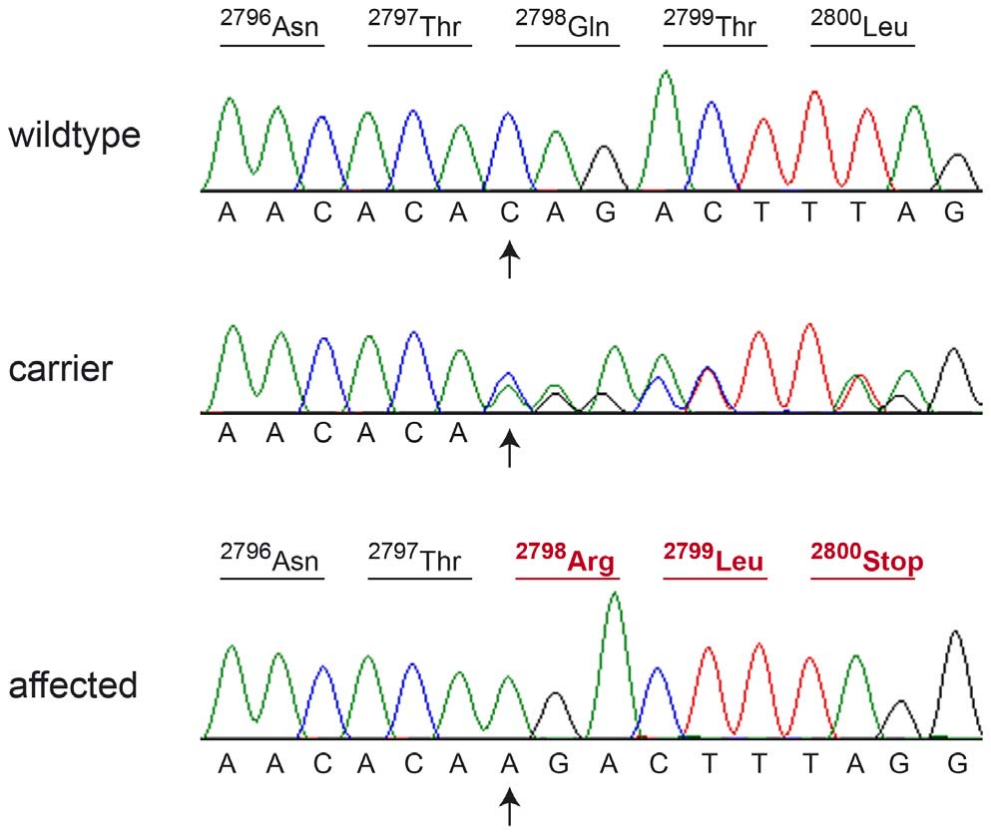
Sanger sequencing of the *CUBN*:c.8392delC variant. Electropherograms of a homozygous wildtype, heterozygous, and homozygous mutant dog, respectively, are shown. The position of the deletion is indicated by arrows. The predicted amino acid translation is shown above the sequence. Altered codons in the affected dog are shown in red. The deletion results in an early premature stop codon (p.Q2798Rfs*3).

We genotyped all remaining non-synonymous variants in larger cohorts of dogs (Table 3). Two variants, *MRC1:c.2143C>T* and *CUBN:c.8392delC*, were perfectly associated with IGS in a cohort of 200 Border Collies. These variants were absent from more than 300 dogs of other breeds. The *MRC1:c.2143C>T* variant represents a nonsense mutation and is predicted to truncate more than 50% of the mannose receptor, C type 1 (p.R715*). The *CUBN:c.8392delC* variant is predicted to result in a frameshift and early premature termination codon in the open reading frame encoding cubilin (p.Q2798Rfs*3; Figure 2).

**Table 3:** Association of non-synonymous variants with the IGS phenotype.

Genotype	Border Collie cases	Border Collie controls	Dogs from other breeds
*MRC1:c.2143C>T* a			
* C/C*	–	175	306
* C/T*	–	12	–
* T/T*	7	–	–
***CUBN:c.8392delC*** a			
*** C/C***	–	181	357
*** C/del***	–	12	–
*** del/del***	7	–	–
*CUBN:c.9215G>C*			
* G/G*	–	64	218
* G/C*	–	87	93
* C/C*	7	85	16

a We observed perfect linkage disequilibrium between the *MRC1:c.2143C>T* and *CUBN:c.8392delC* variants in a sample of 557 dogs. The slightly varying numbers in the table are due to some failed genotyping assays. A list of the control dogs and their respective breeds is given in Table S3.

## Discussion

Using a purely positional approach, we have identified two variants that are perfectly associated with IGS in Border Collies. Both of these variants in the *MRC1* and *CUBN* genes, respectively, lead to premature stop codons and are predicted to completely abolish the function of the encoded proteins. The *MRC1* gene encodes the mannose receptor, C type 1, which is expressed on macrophages and endothelial cells of the liver. MRC1 has a presumed role in the immune system by acting as an essential regulator of inflammation-related serum glycoproteins [16]. *Mrc1* deficient mice have no obvious phenotype other than elevated serum lysosomal enzymes related to slower clearance of serum glycoproteins, including the acid hydrolases [16], [17].

The *CUBN* gene encoding cubilin on the other hand has a well established role in cobalamin uptake. Mutations in this gene have been shown to cause IGS, also termed megaloblastic anemia 1 in humans (MGA1, OMIM #261100) [4]. In contrast to *MRC1*, *CUBN* is thus an excellent functional candidate gene for the hereditary cobalamin malabsorption disorder observed in Border Collies. We therefore conclude that the *CUBN*:c.8392delC variant is the most likely causative defect for the phenotype, which we propose to call IGS in analogy to the human disease. According to our knowledge, these are the first dogs with a characterized molecular defect in the *CUBN* gene. Together with *AMN* mutant Australian Shepherd Dogs and Giant Schnauzers [7] the *CUBN* mutant Border Collies now complete the repertoire of molecular characterized dog models for IGS in humans.

The carrier frequency of dogs being heterozygous for the *CUBN*:c.8392delC variant in a cohort of nearly 200 randomly selected Border Collies is relatively low at 6.2%.

This study highlights the power of complete genome re-sequencing. Using this approach we were able to quickly identify the most likely causative variant for IGS in Border Collies. This variant could also have been obtained by a conventional candidate gene approach. In the case of IGS however, the two known candidate genes would have comprised a total of 79 exons, and the individual design of PCR primers and traditional workflow of PCR-amplifying each exon followed by Sanger sequencing would have required about the same amount of time and money in our laboratory as the whole genome re-sequencing experiment. In contrast to data from a conventional and more limited candidate gene approach, our analysis has also revealed the unexpected finding of a nonsense mutation in the *MRC1* gene. The *MRC1* variant is located 849 kb away from the *CUBN* frameshift variant on dog chromosome 2. Both variants appear to segregate only in Border Collies and have thus most likely arisen within the last 200 years. Unfortunately, the *MRC1* and *CUBN* variants were in perfect linkage disequilibrium in all available dogs of our study, so that we could not disentangle the functional effects of each variant separately. Nonetheless, the seven IGS affected Border Collies did not show any obvious differences in their clinical phenotype with respect to other dogs with primary cobalamin absorption disorders. In particular they showed no signs of immunodeficiency or exaggerated inflammatory reactions (data not shown). It therefore appears that a spontaneous inactivation of the *MRC1* gene in dogs does not result in any obvious clinical phenotype, similar to what has been observed in *Mrc1* knock out mice [16].

In conclusion the identification of a candidate causative mutation for IGS in Border Collies provides the first dog model for IGS with a molecular characterized CUBN defect. Our findings will allow the development of a genetic test and eradication of IGS from the privately owned Border Collie breeding population.

## Materials and Methods

### Ethics Statement

All animal experiments were performed according to the local regulations. The dogs in this study were examined with the consent of their owners. The study was approved by the “Cantonal Committee For Animal Experiments” (Canton of Bern; permits 22/07 and 23/10).

### Animal Selection

We used 7 Border Collie cases, which could be unambiguously phenotyped based on small growth, poor BCS, undetectable serum cobalamin concentrations, methylmalonic aciduria, homocysteinemia (4/7) and complete clinical response to exclusive parenteral cobalamin supplementation. These were all affected dogs that we could obtain for the study and thus represent a convenience sample. Two of the cases were full-siblings. The 7 control Border Collies for the GWAS were judged to be healthy based on unremarkable history and physical examination, as well as normal normal results of CBC, serum biochemistry, urinalysis, serum cobalamin, urinary methylmalonic acid, and plasma homocysteine concentration. We classified additional dogs as controls based on owner-reported unremarkable histories. The complete cohort for this study consisted of 200 Border Collies and 357 dogs of diverse other breeds (Table S3). We collected EDTA blood samples from all dogs.

### DNA Samples and SNP Genotyping

We isolated genomic DNA samples from EDTA blood with the Nucleon Bacc2 kit (GE Healthcare). Genotyping was done on illumina canine_HD chips containing 173,662 SNP markers at the NCCR Genomics Platform of the University of Geneva. Genotypes were stored in a BC/Gene database version 3.5 (BC/Platforms).

### Genome-wide Association Study (GWAS) and Homozygosity Mapping

We used PLINK v1.07 [18] to perform genome-wide association analyses (GWAS). We removed markers and individuals with call rates <90% from the analysis. We also removed markers with minor allele frequency (MAF) <5% and markers strongly deviating from Hardy-Weinberg equilibrium (p<10^−5^). We performed an allelic association study using the –assoc command of PLINK. We also used PLINK to search for extended intervals of homozygosity with shared alleles as described previously [19].

### Statistics

Raw p-values in the GWAS are based on χ^2^ tests of the allele frequency in cases vs the allele frequency in controls for each marker. After the filtering procedures described above, we had 112,323 markers left for the final analysis. In order to correct for the multiple testing situation, we determined an empirical significance threshold by performing 100,000 permutations of the dataset with arbitrarily assigned phenotypes.

### Gene Analysis

We used the dog CanFam 3 genome assembly derived from a Boxer as reference genome sequence. All numbering within the canine *CUBN* gene corresponds to the accessions NM_001003148.1 (mRNA) and NP_001003148.1 (protein).

### Whole Genome Sequencing of an Affected Border Collie

We prepared a fragment library with 300 bp insert size and collected one lane of illumina HiSeq2000 paired-end reads (2×100 bp). We obtained a total of 125,457,731 paired-end reads or roughly 10× coverage. We mapped the reads to the dog reference genome with the Burrows-Wheeler Aligner (BWA) version 0.5.9-r16 [20] with default settings and obtained 243,130,060 uniquely mapping reads. After sorting the mapped reads by the coordinates of the sequence with Picard tools, we labeled the PCR duplicates also with Picard tools (http://sourceforge.net/projects/picard/). We used the Genome Analysis Tool Kit (GATK version 0591, [21]) to perform local realignment and to produce a cleaned BAM file. Variants calls were then made with the unified genotyper module of GATK. Variant data for each sample were obtained in variant call format (version 4.0) as raw calls for all samples and sites flagged using the variant filtration module of GATK. Variant calls that failed to pass the following filters were labeled accordingly in the call set: (i) Hard to Validate MQ0≥4 & ((MQ0/(1.0 * DP)) >0.1); (ii) strand bias (low Quality scores) QUAL <30.0 || (Quality by depth) QD <5.0 || (homopolymer runs ) HRun >5 || (strand bias) SB >0.00; (iii) SNP cluster window size 10. The snpEFF software [22] together with the CanFam 3.1 annotation was used to predict the functional effects of detected variants.

### Sanger Sequencing

We used Sanger sequencing to confirm the illumina sequencing results and to perform targeted genotyping for selected variants. For these experiments we amplified PCR products using AmpliTaqGold360Mastermix (Applied Biosystems). PCR products were directly sequenced on an ABI 3730 capillary sequencer (Applied Biosystems) after treatment with exonuclease I and shrimp alkaline phosphatase. We analyzed the sequence data with Sequencher 4.9 (GeneCodes).

## Supporting Information

Table S1 **Genes in the 3.53 Mb critical interval on CFA 2.**
(XLSX)

Table S2 **Non-synonymous and associated variants in the 3.53 Mb critical interval on CFA 2.**
(XLSX)

Table S3 **List of control dogs and breeds that were used for the association study.**
(XLSX)
